# Tension Recovery following Ramp-Shaped Release in High-Ca and Low-Ca Rigor Muscle Fibers: Evidence for the Dynamic State of AMADP Myosin Heads in the Absence of ATP

**DOI:** 10.1371/journal.pone.0162003

**Published:** 2016-09-01

**Authors:** Haruo Sugi, Maki Yamaguchi, Tetsuo Ohno, Takakazu Kobayashi, Shigeru Chaen, Hiroshi Okuyama

**Affiliations:** 1 Department of Physiology, School of Medicine, Teikyo University, Tokyo, Japan; 2 Department of Physiology, Jikei University School of Medicine, Tokyo, Japan; 3 Department of Electronic Engineering, Shibaura Institute of Technology, Tokyo, Japan; 4 Department of Integrated Sciences in Physics and Biology, College of Humanities and Science, Nihon University, Tokyo, Japan; University of Debrecen, HUNGARY

## Abstract

During muscle contraction, myosin heads (M) bound to actin (A) perform power stroke associated with reaction, AMADPPi → AM + ADP + Pi. In this scheme, A • M is believed to be a high-affinity complex after removal of ATP. Biochemical studies on extracted protein samples show that, in the AM complex, actin-binding sites are located at both sides of junctional peptide between 50K and 20K segments of myosin heavy chain. Recently, we found that a monoclonal antibody (IgG) to the junctional peptide had no effect on both in vitro actin-myosin sliding and skinned muscle fiber contraction, though it covers the actin-binding sites on myosin. It follows from this that, during muscle contraction, myosin heads do not pass through the static rigor AM configuration, determined biochemically and electron microscopically using extracted protein samples. To study the nature of AM and AMADP myosin heads, actually existing in muscle, we examined mechanical responses to ramp-shaped releases (0.5% of Lo, complete in 5ms) in single skinned rabbit psoas muscle fibers in high-Ca (pCa, 4) and low-Ca (pCa, >9) rigor states. The fibers exhibited initial elastic tension drop and subsequent small but definite tension recovery to a steady level. The tension recovery was present over many minutes in high-Ca rigor fibers, while it tended to decrease quickly in low-Ca rigor fibers. EDTA (10mM, with MgCl_2_ removed) had no appreciable effect on the tension recovery in high-Ca rigor fibers, while it completely eliminated the tension recovery in low-Ca rigor fibers. These results suggest that the AMADP myosin heads in rigor muscle have long lifetimes and dynamic properties, which show up as the tension recovery following applied release. Possible AM linkage structure in muscle is discussed in connection with the X-ray diffraction pattern from contracting muscle, which is intermediate between resting and rigor muscles.

## Introduction

Muscle contraction results from relative sliding between actin and myosin filaments, coupled with ATP hydrolysis [1,2], which in turn is produced by attachment-detachment cycle between the myosin head extending from myosin filaments and the sites on actin filaments. On the basis of actomyosin ATPase reaction steps in solution [3], myosin head (M), in the form of M • ADP • Pi first attaches to actin (A), and performs a power stroke, associated with reaction, AMADPPi withtin (A), and so that at the end of power stroke, M takes the form AM, i.e. rigor (or rigor-like) configuration. Upon binding with a new ATP, M detaches from A to perform a recovery stroke, associated with reaction, MATP performn head extendintaches to A. In this scheme, it is generally believed that the AM corresponds to a high-affinity complex between actin and myosin head in the absence of ATP, i.e. the AM complex present in rigor muscle. The rigor AM complex has been well characterized biochemically using extracted protein samples [4] and visualized electron microscopically as acto-S1 complex, around which isolated single myosin heads (S1) are attached [5]. Based on the static rigor AM linkages, it is implicitly believed that, in rigor fibers, tension is only passively maintained.

Contrary to the general view stated above, however, we have recently found that a monoclonal antibody to the 50K-20K junctional peptide in myosin has no effect on both ATP-dependent in vitro actin myosin sliding and Ca^2+^-activated skinned muscle fibers [6], though the antibody (IgG) covers actin-binding sites at both sides of the junctional peptide [7]. This indicates that, in the attachment-detachment cycle between myosin heads and actin, myosin heads do not take the well established rigor AM configuration, determined biochemically and electron microscopically using extracted protein samples [5,6]. In addition, it has long been known that the X-ray diffraction pattern from contracting muscle is intermediate between those from relaxed and rigor muscles, though a considerable proportion of myosin heads in contracting muscle are strongly bound to actin [8].

To obtain information about the nature of strong AM linkages, constituting AM and AMADP in contracting muscle [7], we have studied mechanical response of skinned rabbit psoas muscle fibers to ramp-shaped release in both high-Ca and low-Ca rigor states; the former state is established by removing external ATP from contracting solution (pCa, 4), while the latter state is attained by removing external ATP from relaxing solution (pCa,>9). We assume that, in high-Ca rigor solution, a large proportion of myosin heads are put into rigor state after performing their last power stroke, thus more or less preserving their configuration at the end of power stroke. In low-Ca rigor solution, on the other hand, all myosin heads, initially detached from actin, have to bind with actin by overriding tropomyosin around actin filaments, thus taking variable rigor configurations. Based on the above assumptions, we expected to find definite differences in the mechanical response to applied release between high-Ca and low-Ca rigor muscle fibers.

Here we report that, in response to ramp-shaped releases (amplitude, 0.5% of Lo or ~6nm/half sarcomere; duration, 5ms), the fibers in both high-Ca and low-Ca rigor states first exhibit elastic drop in rigor tension coincident with the applied release, and then exhibit small but distinct redevelopment of rigor tension (tension recovery) to a steady level. In high-Ca rigor fibers, the tension recovery following ramp-shaped releases was observed over many minutes, and was not affected appreciably in the presence of EDTA (external MgCl_2_ removed). In contrast, in low-Ca rigor fibers, the tension recovery tended to decrease rapidly, and was completely eliminated in the presence of EDTA. These findings indicate that the AMADP myosin heads in rigor muscle have long lifetimes and dynamic properties, which show up as the tension recovery following applied release.

## Materials and Methods

### Skinned Muscle Fiber Preparation and Experimental Setup

Eight white male rabbits weighing 2tionim (Japan White, Sankyo Lab. Industry) were killed on their delivery to our laboratory by sodium pentobarbital injection (50mg/kg) into ear vein, and psoas muscles were dissected from the animals. The animals treated following the Guiding Principles for the Care and Use of Animals in the Field of Physiological Sciences, published by the Physiological Society of Japan. The protocol was approved by the Teikyo University Animal Care Committee (protocol #07–050). The animals, delivered from a chemically skinned muscle fiber strips were prepared from the psoas muscle as described by Sugi et al. [9]. Single muscle fibers (diameter, 40–60**μ**m) were isolated from the fiber strips, and mounted horizontally in an experimental apparatus between a force transducer (AE801, SensoNor, Holten, Norway) and a servomotor (G-100PD, General Scanning, Watertown, MA) by glueing both ends with collodion. The servomotor contained a displacement transducer (differential capacitor) sensing the motor arm movement. Further details of the experimental apparatus have described elsewhere [9,10]. The fiber was kept at its slack length Lo (~3mm) at a sarcomere length of 2.4**μ**m, measured with optical diffraction by He-Ne laser light. The experimental apparatus consisted of five solution cells (volume, 100–(vo**μ**l for each) made of anodized aluminum blocks [10]. Exchange of solutions were made by lifting the fiber up from one compartment, and then putting it into another compartment. Experiments were made at 20°C.

### Solutions

Relaxing solution (pCa, >9) contained 125mM KCl, 4mM MgCl_2_, 4mM ATP, 4mM EGTA, and 20mM PIPES. Contracting solution (pCa, 4) was prepared by adding 4mM CaCl_2_ to activate the fibers maximally. High-Ca rigor solution (pCa, 4) was prepared by omitting ATP from contracting solution, while low-Ca rigor solution was prepared by omitting 4mM ATP from relaxing solution. In all solutions, pH was adjusted to 7.0 with PIPES. When EDTA (10mM) was added to solutions, MgCl_2_ was removed, and the ionic strength of solutions was kept approximately 170mM by changing KCl concentration. In some experiments, hexokinase (50 unit/ml) and D-glucose (2mM) were added to the rigor solution to facilitate removal of ATP with similar results.

### Recording of Muscle Fiber Stiffness with Sinusoidal Vibrations

To estimate the time required to establish rigor state after putting the fibers into rigor solutions, we recorded changes in muscle fiber stiffness by applying small sinusoidal vibrations (peak-to-peak amplitude, 0.2% of slack fiber length Lo; frequency, 2kHz) with the servo-motor [9,11]. The tension signals consisted of muscle fiber tension and superimposed sinusoidal component in response to applied vibration. The in-phase sinusoidal component (in-phase stiffness) and the (90deg) out-of-phase component (quadrature stiffness) were separated from muscle fiber tension with a lock-in amplifier to be recorded together with tension changes in the fiber. All the experimental records were displayed and recorded on an X-Y chart recorder [9–12].

### Application of Ramp-Shaped Releases to Rigor Muscle Fibers

To obtain information about changes in the state of myosin heads when the fibers were put into rigor state, we applied ramp-shaped releases (amplitude, 0.5% of Lo or ~6nm/half sarcomere; duration, 5ms) to the fibers with the servo-motor [9]. The length change signal was produced with a waveform generator (Wavetech, model 164). After each application of ramp-shaped releases, the fiber was restretched slowly to the initial length in 3–4s, to avoid stretch-induced damage to rigor fibers [13]. A series of release-restretch cycle were applied to the fiber at intervals of 30ints, so that tension responses of the fibers to ramp-shaped releases were recorded at various times after transfer of the fiber into rigor solution. Experimental data were acquired in a microcomputer (PC-9801A, NEC) through an analog-to-digital converter of 12bit resolution, and displayed on an X-Y plotter after appropriate filtering to remove high frequency noise [9].

## Results

### Estimation of ATP Diffusion Out of and Into Muscle fibers

**Fig 1:**
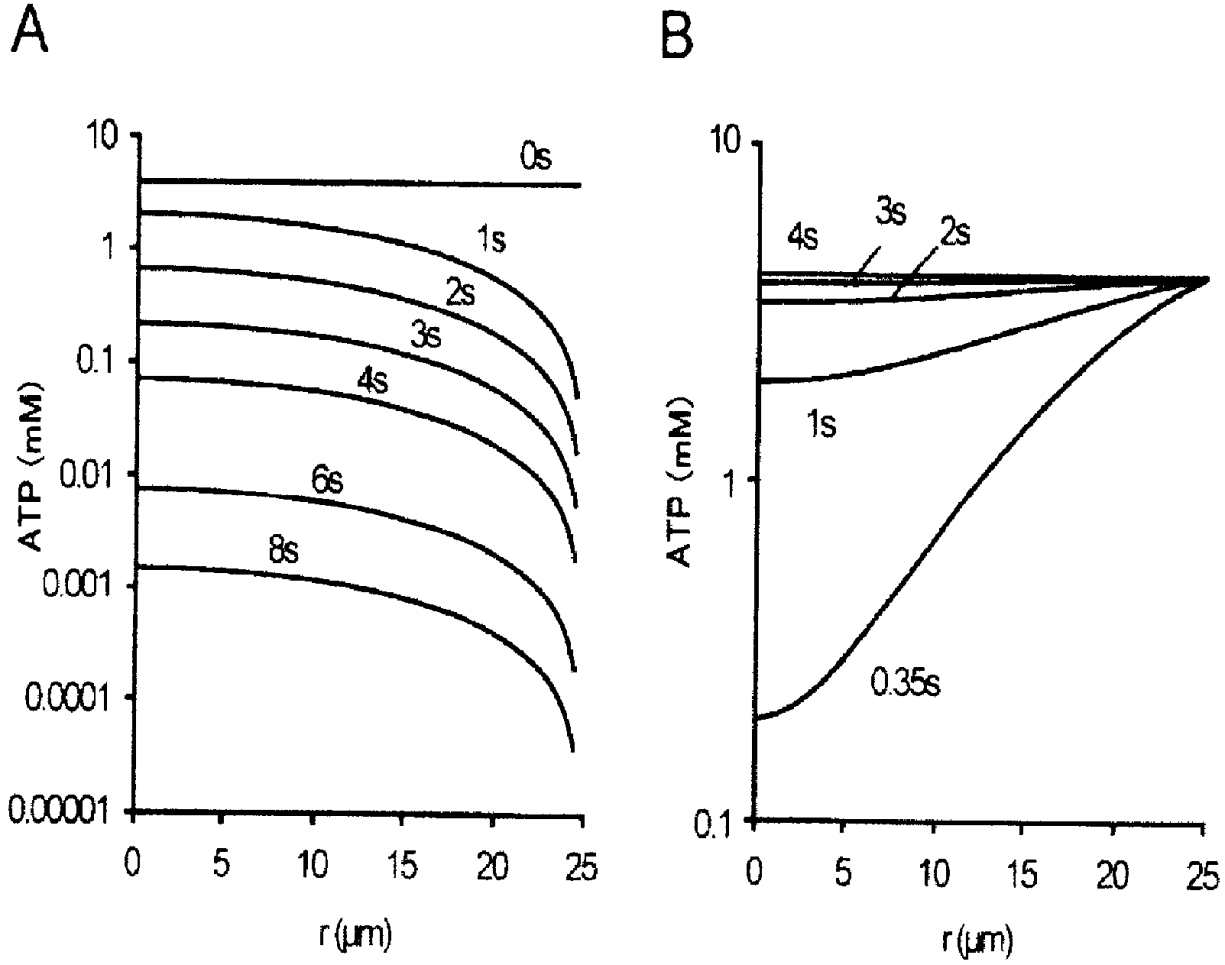
Computed Changes of ATP concentration gradient across the cross-section of a single muscle fiber. (A) Change of ATP concentration *C* gradient at various times after transfer of a single muscle fiber (diameter, 50**μ** m) from contracting solution to ATP-free rigor solution. (B) Change of ATP concentration gradient at various times after transfer of the fiber from rigor solution to relaxing solution containing 4mM ATP. In both A and B, ATP concentrations (mM) in logarithmic scale are plotted against distances *r* (μm) from the center of the fiber. Calculations were performed by the equation, *dC(r) / dt = D [d*^*2*^*C(r) / dr*^*2*^ + 1/*r* • dC(r) / dt, where *C(r)* is ATP concentration as a function of *r*.

Prior to the experiments, we computed concentrations of ATP along the cross-section of a skinned muscle fiber (diameter, 50**μ**m) at various times after the fiber was transferred from contracting solution to rigor solution, or from rigor solution to relaxing solution, assuming the diffusion coefficient of ATP of 1.2 x 10^−6^ cm2 / s) [14]. As shown in Fig 1A, the ATP concentration at the center of the fiber is reduced from 4mM to < 10**μ**M at 6s after putting the fiber into rigor solution. In this calculation, ATPase activity of myosin heads was not taken into consideration. It would therefore be safe to expect that, if the fiber is kept in rigor solution for 10s, ATP concentration at the center of the fiber is reduced to < 1**μ**M, i.e. a negligibly small fraction of myosin head concentration in the fiber (~200**μ**M) [10]. On the other hand, the ATP concentration at the center of the fiber was increased to > 200**μ**M at 0.35s after returning the rigor fibers to relaxing solution containing 4mM ATP (Fig 1B), indicating that all myosin heads may detach from actin well within 1s after transfer of the fiber to relaxing solution.

### Tension and Stiffness Changes Before and After Transfer of the Fibers to High-Ca and Low-Ca Rigor Solution

**Fig 2:**
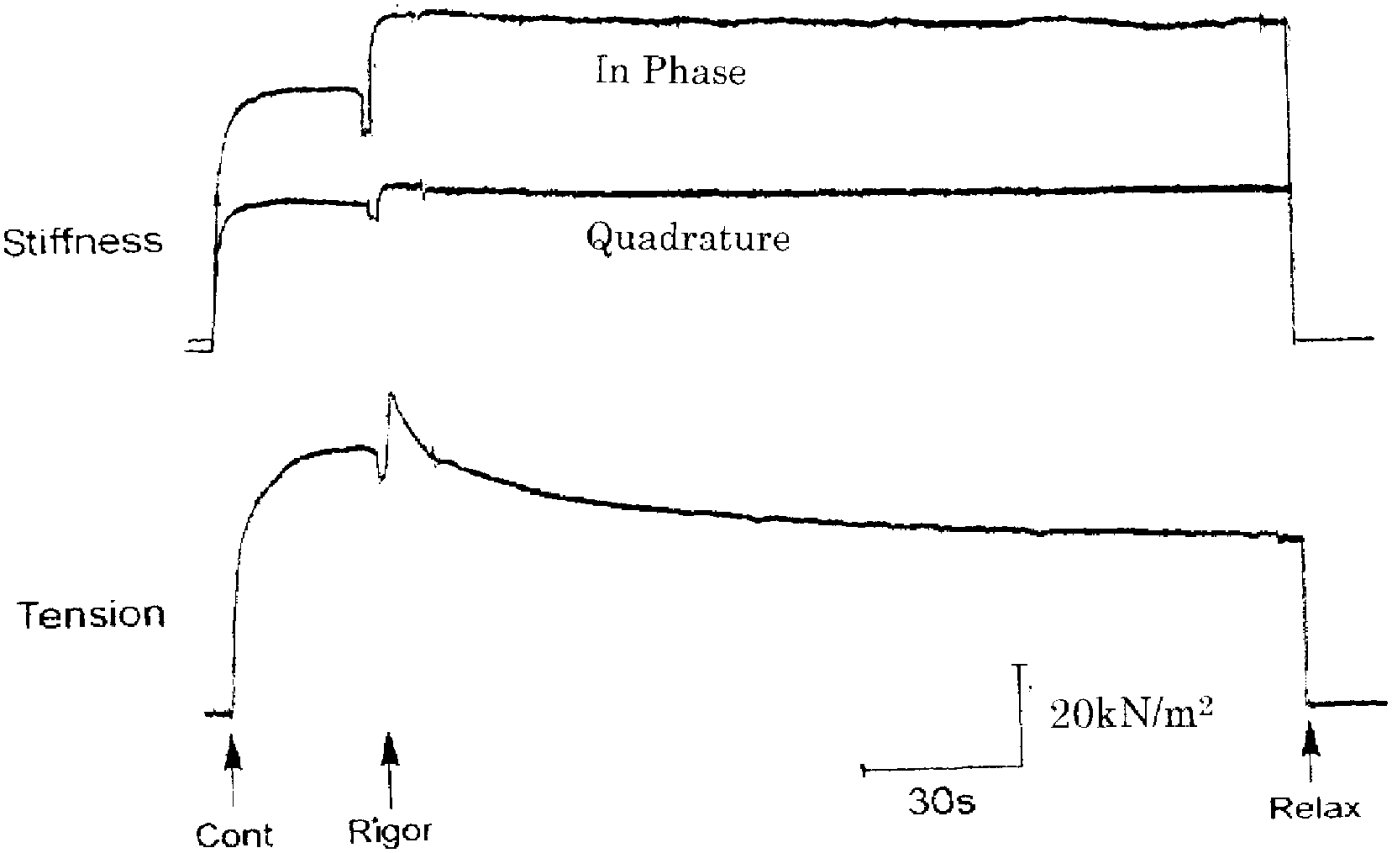
Stiffness and tension changes when a skinned fiber is put into high-Ca^2+^ rigor state. The fiber was first activated maximally in contracting solution, and then transferred into high-Ca rigor solution. Arrows indicate timing of solution exchanges. In this and Fig 3, upper traces show changes in in-phase stiffness and quadrature stiffness, while lower trace shows tension. The tension increment on application of rigor solution is an artefact accompanying solution exchange procedure.

To estimate the time required for establishment of rigor state in muscle fibers, when they were transferred into rigor solutions, we made continuous recording of muscle fiber stiffness. Fig 2 shows changes in stiffness and tension when a single muscle fiber, generating the maximum Ca^2+^-activated isometric tension Po (40–70kN / m^2^), was transferred from contracting solution (pCa, 4) to high-Ca rigor solution (pCa, 4). The tension in the fiber fell by 30–40% from Po for the first few minutes after application of rigor solution, while in-phase stiffness increased by ~30% within 10s after application of rigor solution, indicating that high-Ca rigor state may be established when the ATP concentration at the center of the fiber is reduced to <10**μ**M (Fig 1A). On the other hand, quadrature stiffness increased by ~5% on application of rigor solution.

**Fig 3:**
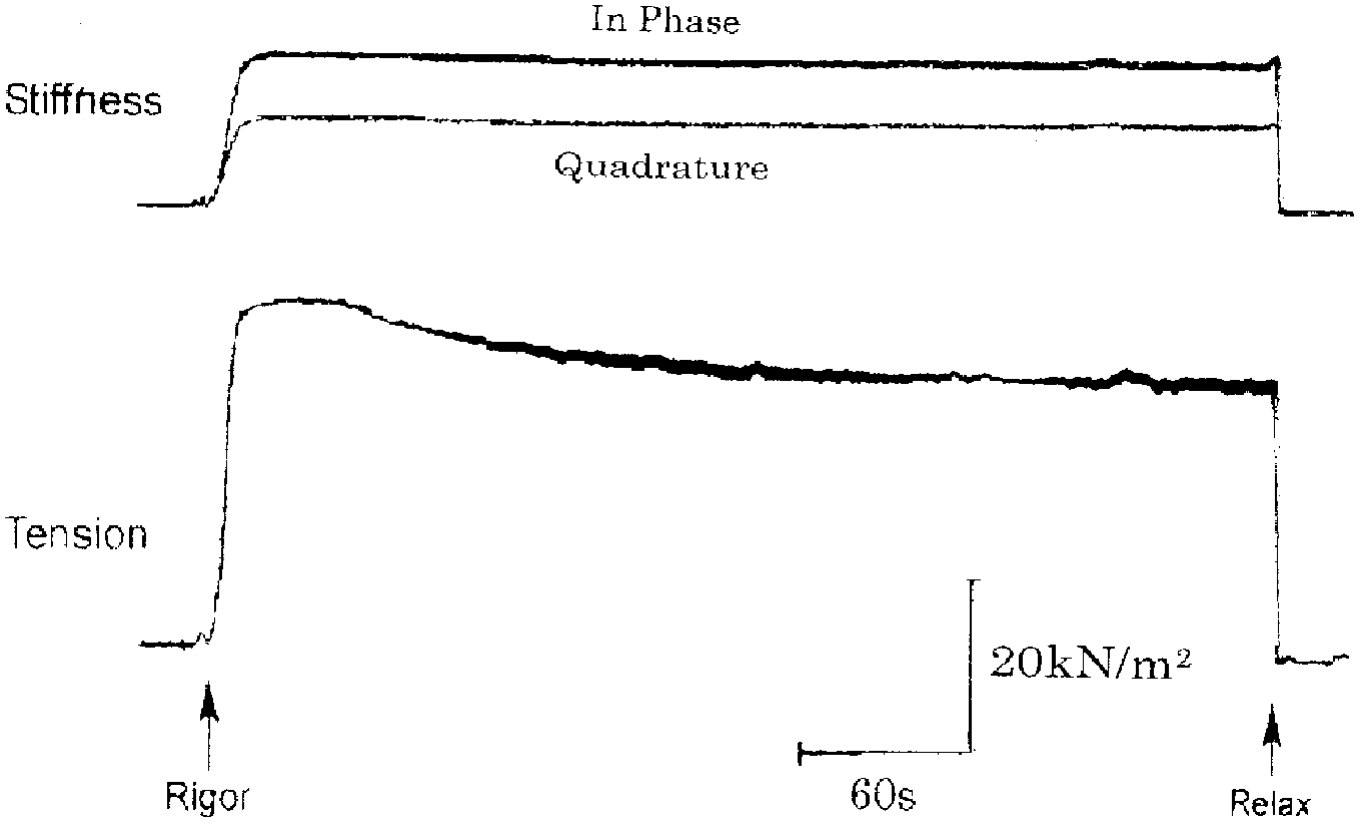
Stiffness and tension changes when a skinned fiber was put into low-Ca rigor state. The fiber was transferred from relaxing solution to low-Ca rigor solution.

Fig 3 shows changes in stiffness and tension when a relaxed single muscle fiber is transferred from relaxing solution (pCa, > 9) to low-Ca rigor solution (pCa, >9). In most fibers examined, both in-phase and quadrature stiffness increased nearly in parallel with increasing low-Ca rigor tension, and reached a peak in ~30s after application of rigor solution. This indicates that low-Ca rigor state is established in ~30s after transfer of the fibers into low-Ca rigor solution. After reaching a peak (20–40kN / m^2^), low-Ca rigor tension decreased with time with variable rate nearly in parallel with stiffness. On returning the fiber in high-Ca or low-Ca rigor states to relaxing solution, both rigor tension and stiffness fell to zero within 1s, being consistent with the calculated rate of ATP diffusion into the fiber (Fig 1B).

### Determination of Amplitude and Velocity of Ramp-Shaped Releases

In the research field of muscle mechanics, important information about molecular mechanism of muscle contraction has been obtained from mechanical response of contracting muscle fibers to quick release, but not from that to quick stretch [15, 16]. In the present study, we also focused attention only on the mechanical response of rigor fibers to ramp-shaped releases, but not stretches.

To apply release-restretch cycles repeatedly to rigor fibers without giving appreciable damage to the fibers, the amplitude of ramp-shaped release has been limited to 0.5% of Lo (~6nm / half sarcomere), being well within the range of distance in which myosin heads can move while kept attached to actin [15]. Releases of shorter duration tended to give damage to rigor fibers, as evidenced by rapid reduction of rigor tension produced by repeated application of release. At 30–40s after each release, the fibers were restretched to the initial length slowly in 2–6s, to prevent rupture of myofilaments caused by rapid stretches [13].

### Tension Recovery in High-Ca Rigor Fibers Following Ramp-Shaped Releases

**Fig 4:**
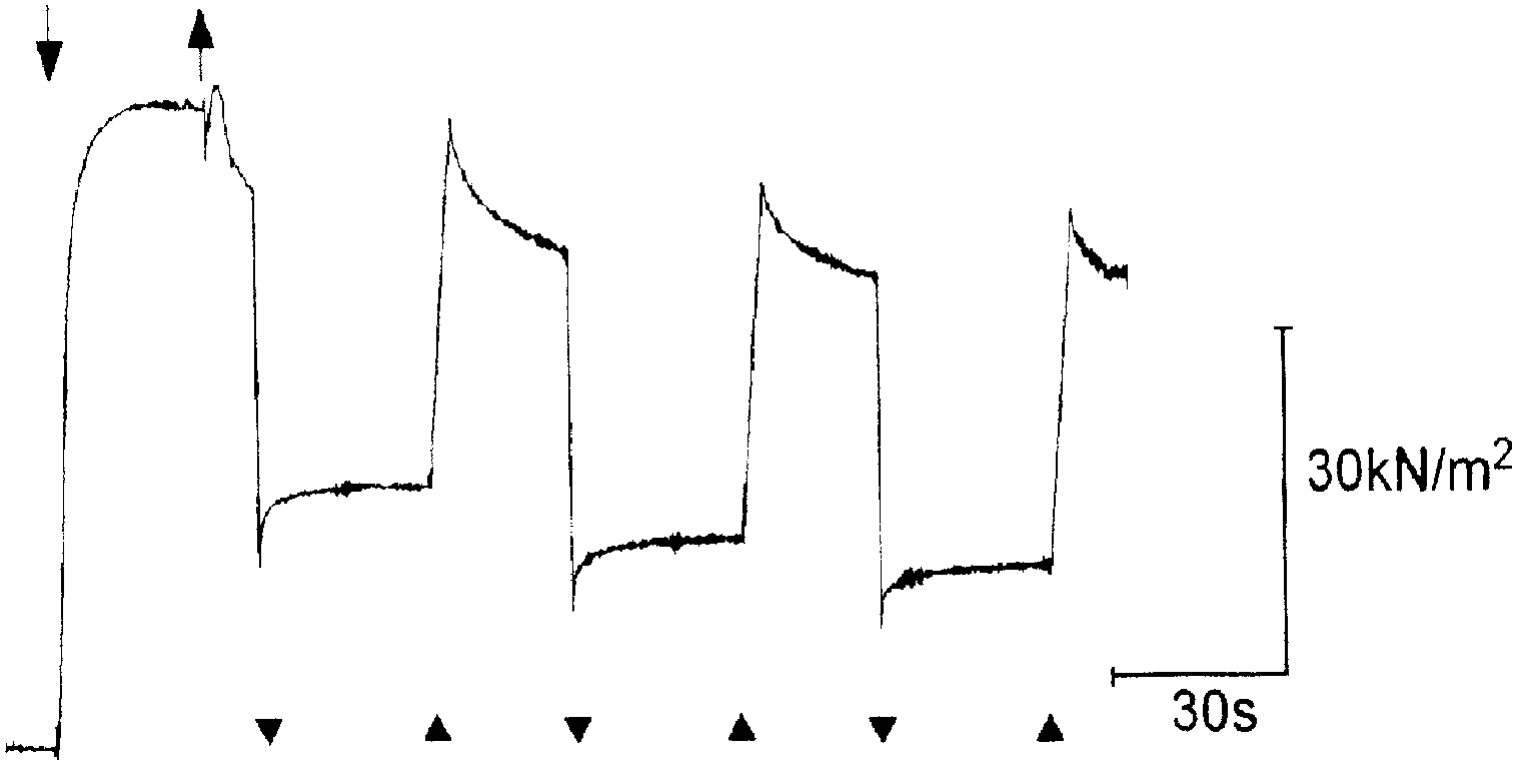
Tension responses of skinned fibers in high-Ca rigor state to repeated application of ramp-shaped releases. Ramp-shaped releases (amplitude, 0.5% of Lo; duration, 5ms) were repeatedly applied to the fibers in high-Ca rigor state at appropriate intervals. Note that, following each release, initial elastic drop of tension is followed by distinct tension redevelopment, i.e. tension recovery to a steady level. Downward and upward arrows indicate times of application of contracting and rigor solutions, respectively. In this and Fig 7, downward and upward arrowheads at the bottom of tension records indicate times of application of release and restretch, respectively.

First, we examined mechanical response of fibers in high-Ca rigor state, which was established by transferring isometrically contracting fibers from contracting solution (pCa, 4) to high-Ca rigor solution (pCa, 4). As already mentioned, rigor myosin heads are expected to establish after performing their last power stroke, without being inhibited by tropomyosin around actin filaments. Fig 4 shows an examples of tension records in the fibers in high-Ca rigor state, to which ramp-shaped releases (amplitude (0.5% of Lo), each followed by restretch, were repeatedly applied. The fibers were restretched at 30–40s after each release, and subjected to next release at 30–40s after completion of preceding restretch. In response to ramp-shaped release, rigor fibers exhibited initial elastic tension drop coincident with applied release, which was followed by small but distinct tension redevelopment to steady level. Time course of tension redevelopment was exponential in shape, approaching a steady level asymptotically. The half rise time of tension redevelopment showed a wide range of variation, ranging from 1 to > 5s.

The tension redevelopment in rigor fibers resembled in appearance that of quick tension recovery following quick release in contracting muscle fibers [15], though the time scale was ~three orders of magnitude slower in the former than in the latter. In the present paper, the tension redevelopment in rigor fibers will be called tension recovery. As can be seen in Fig 4, the tension rose to a peak during restretch of the fibers to the initial length, and after completion of restretch decayed exponentially with time. The asymmetric tension responses in rigor fibers with respect to direction of applied length changes are also analogous to those in contracting fibers.

### Characterization of Tension Recovery in High-Ca Rigor Fibers

**Fig 5:**
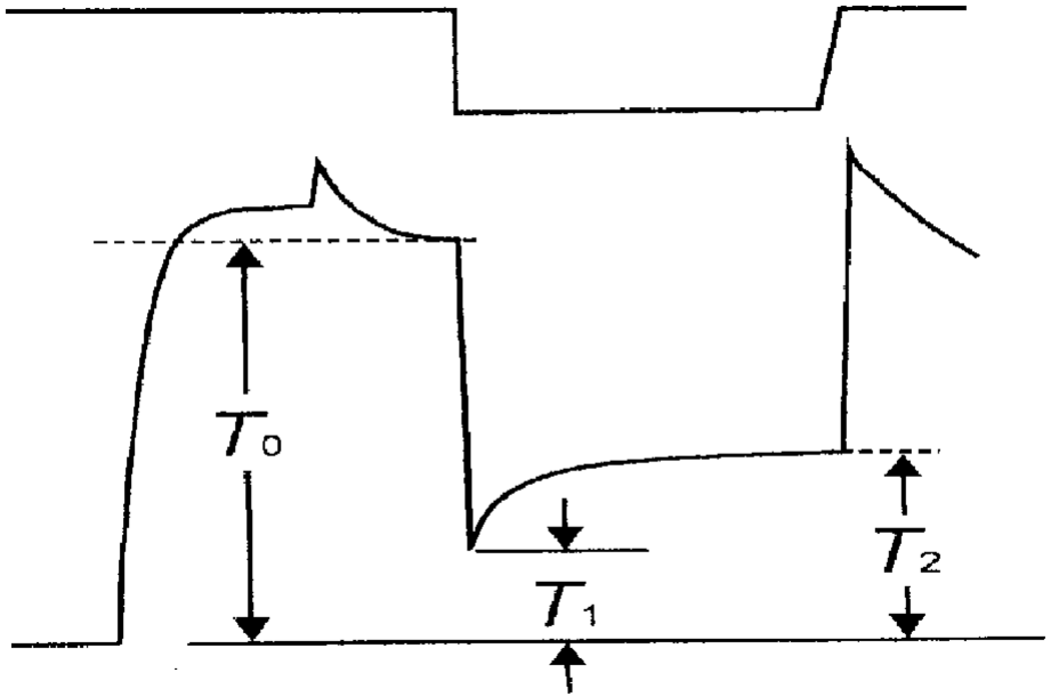
Diagram illustrating method of estimating amplitude of tension recovery. Upper and lower traces show length and tension changes of the fiber, respectively. The fiber is activated maximally in contracting solution, and transferred to high-Ca rigor solution. On application of release, the initial elastic tension drop from To to T_1_ is followed by the subsequent tension recovery from T_1_ to T_2_. The amplitude of tension recovery relative to To is expressed as Trec = (T_2_–s_1_) / To.

Fig 5 illustrates the method of determining the amplitude of tension recovery in rigor fibers. The tension immediately before release is defined as To. During release, the tension in rigor fibers drops from To to T_1_, and then starts rising towards a steady level T_2_. The amplitude of tension recovery Trec is expressed relative to To, as Trec = (T_2_ –T_1_) / To. In most fibers, tension rose from T_1_ to steady level T_2_ in 30–40s, but in some fibers tension was still rising toward a steady level; in such fibers, we determined approximate value of T_2_ as tension level reached in 30s after the completion of release. The value of Trec was always maximum for the first release, which was applied at 10–15s after transfer of the fibers to rigor solution, and gradually decreased with time in rigor solution. The value of To for the first release was 3–15% smaller than Po. In 28 muscle fibers, generating Po of 50–80kN/m^2^ (20°C), the average value of Trec was 0.14 ± 0.05 (mean ± SD, n = 25). In most high-Ca rigor fibers examined, the amplitude of initial elastic tension drop (To–T_1_) relative to To, i.e. (To–T_1_)/To, serving as a measure of apparent rigor fiber stiffness, ranged from 0.5 to 0.7, and was always smallest following the first release compared to the subsequent releases.

**Fig 6:**
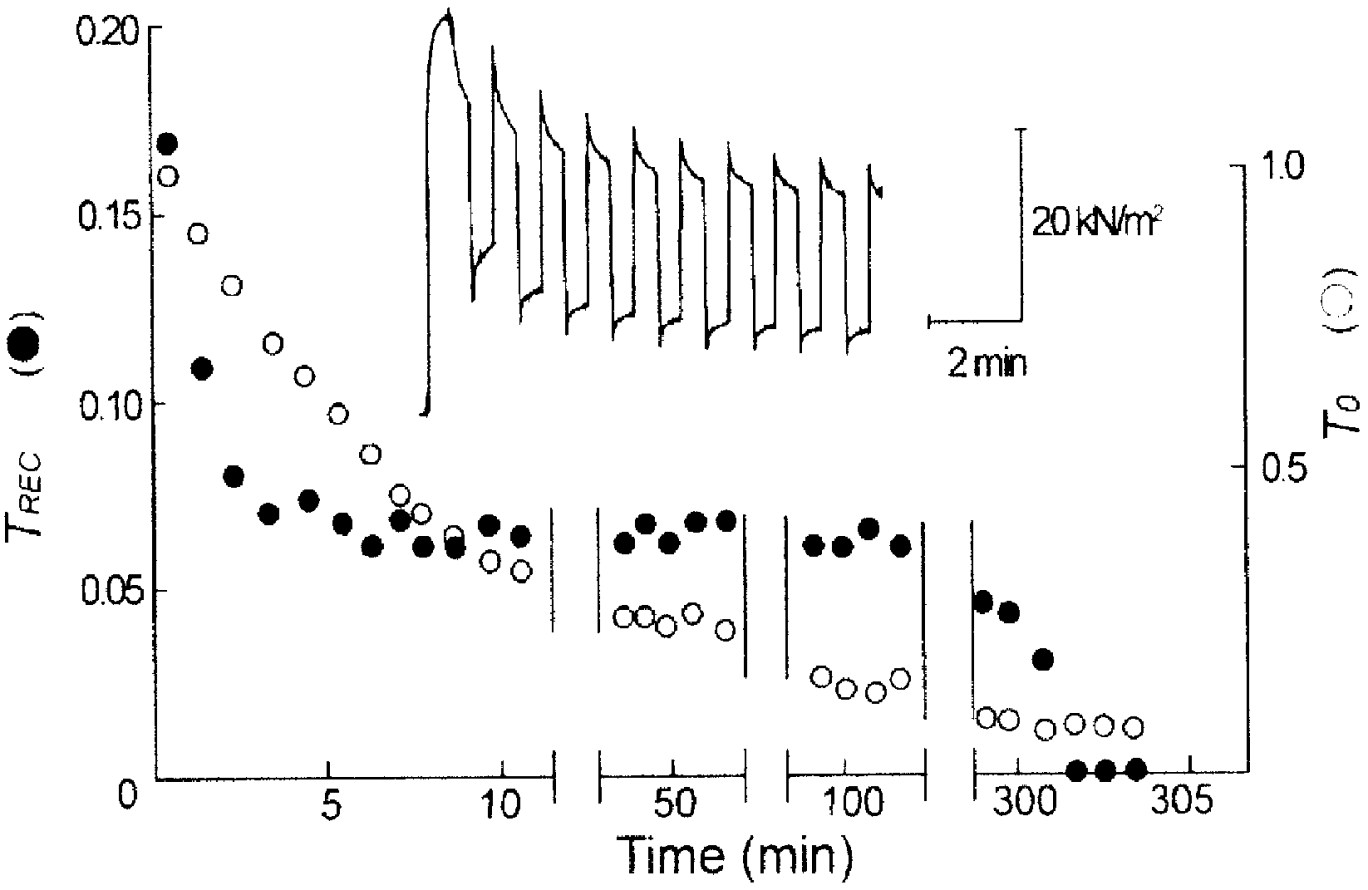
Changes in amplitude of tension recovery and tension immediately before release in high-Ca rigor solution. The fiber was subjected to repeated releases. Values of Trec (filled circles) and the tension immediately before release To (open circles) are plotted against time in high-Ca rigor solution. Tension changes in the fiber in response to the first to the ninth release-restretch cycles are shown in the inset. Note that Trec decreases by ~65% for the first 10min in rigor solution, and then remains almost unchanged over many minutes until it eventually disappears, while To decreases continuously with time in rigor solution.

In Fig 6, values of Trec (filled circles) and To (open circles), determined by repeated application of release-restretch cycles (Inset), are plotted against time in high-Ca^2+^-rigor solution. Trec decreased by ~65% for the first 10min in rigor solution, then remained almost unchanged over subsequent 200min. Trec eventually disappeared, when To decreased to < 10% of the initial value. Similar results were obtained on 5 other fibers examined. To ascertain the possibility that the presence of tension recovery over long periods of time in high-Ca rigor fibers might result from incomplete removal of ATP from the fibers, we examined the effect of EDTA (10mM, with MgCl_2_ removed), chelating Mg ions, on tension recovery. For this purpose, 8 muscle fibers were first kept in EDTA relaxing solution for 5–fomin, made to contract in EDTA contracting solution, and then put into rigor state with EDTA rigor solution. All the fibers examined, exhibited tension recovery similar to that in the fibers not treated with EDTA; the average value of Trec for the first release was 0.15 ± 0.08 (mean ± SD, n = 8), being not significantly different from the corresponding value in the absence of EDTA. This result indicate that the long lasting tension recovery in high-Ca rigor state may not be due to incomplete removal of ATP from the interior of the fibers.

The long lasting tension recovery over many minutes may be accounted for in the following way: (1) The linkage between A and MADP in rigor fibers have a lifetime of many seconds; (2) If MADP myosin heads dissociate from actin, then would again bind with actin while relative position between them does not change appreciably; (3) As the result, AMADP myosin heads exist in rigor fibers over many minutes to produce the tension recovery. The gradual decline of To may result from gradual decrease in the overall strain within rigor fibers as myosin heads repeat detachment from, and reattachment to, actin.

### Tension Recovery in Low-Ca rigor State Fibers Following Ramp-Shaped Releases

Next, we examined mechanical response to ramp-shaped releases in the fibers in low-Ca rigor state, which was established by transferring relaxed fibers from relaxing solution (pCa, >9) into low-Ca rigor solution (pCa, >9). As myosin head binding to actin is inhibited by tropomyosin around actin filaments in relaxed fibers, myosin heads have to override tropomyosin to form rigor AM linkages. Time course of establishment of low-Ca rigor state can be visualized by the development of low-Ca rigor tension after transferring the fibers from relaxing to low-Ca rigor solution.

**Fig 7:**
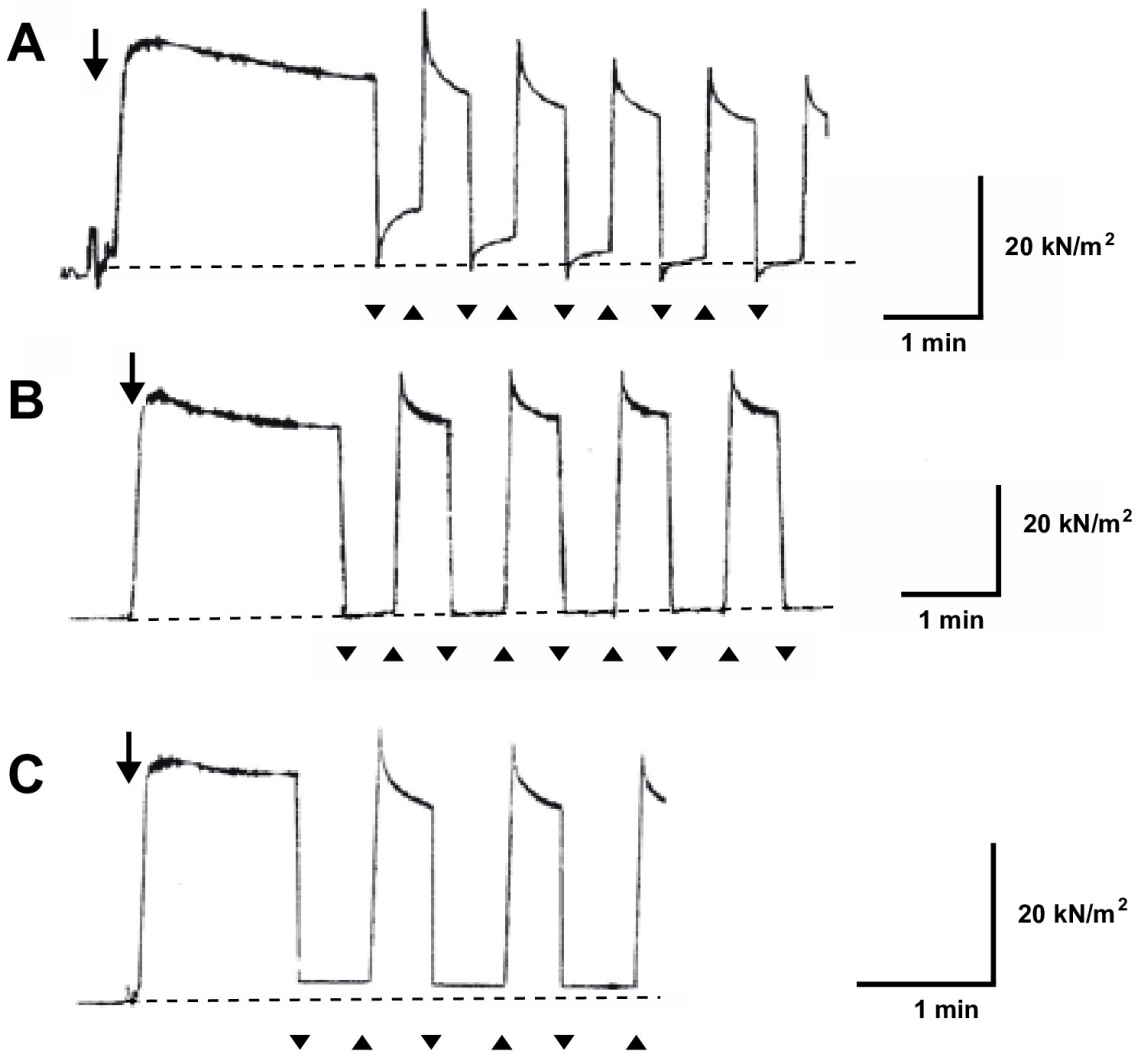
Tension response of skinned fibers in low-Ca rigor state. (A) Tension recovery following repeated releases (amplitude, 0.5% of Lo; duration, 5ms). Note that tension recovery takes place from negative tension below zero tension baseline. (B) Disappearance of tension recovery following repeated releases (amplitude, 0.5% of Lo; duration, 5ms) in the presence of 10mM EDTA. Note that the tension drop coincident with applied release is not followed by tension recovery. (C) Disappearance of tension recovery following repeated releases (amplitude, 0.3% of Lo; duration, 5ms) in the presence of 10mM EDTA. In A, B and C, downward arrow indicates time of application of Low-Ca rigor solution (pCa, > 9), while horizontal broken line indicates zero tension baseline.

When the fibers in low-Ca rigor state were subjected to ramp-shaped releases after development of peak low-Ca rigor tension (amounting ~one third of maximum Ca^2+^-activated tension Po) [17], they also exhibited tension recovery similar to that in high-Ca rigor state, as shown in Fig 7A. The maximum Trec values, obtained at 10–15s after transfer of the fibers to rigor solution, was 0.12 ± 0.06 (mean ± SD, n = 20). In contrast with high-Ca rigor fibers, the value of Trec tended to decrease much more rapidly after each application of release-restretch cycles; reaching almost to zero after application of 5–20 release-restretch cycles. On the other hand, the value of (To–T_1_)/To, representing apparent muscle fiber stiffness, was close to unity for the first release, and tended to increase above unity for the subsequent releases. It can be seen in Fig 7A that the tension drops from To to T_1_ = 0 for the first release, so that (To–T_1_)/To = 1. Following subsequent releases, tension drops from To to T_1_< 0, so that (To–T_1_)/To is >1. Since rigor linkages are very tight, rigor fibers are bent at the end of excessive release to push force transducer to exert negative tension. The slight bending of the fibers did not, however, affect subsequent tension recovery; as can be seen in Fig 7A, the time course of tension recovery tension recovery did not change appreciably despite the increasing amount of negative tension with repeated releases. This indicates that the slight bending of rigor fibers does not appreciably affect tension recovery.

On the other hand, if the fibers were put into low-Ca rigor state in rigor solution containing 10mM EDTA (with MgCl_2_ removed), and subjected to release-restretch cycles after development of peak rigor tension, they did not exhibit tension recovery. As can be seen in Fig 7B, the value of (To–T_1_)/To for the first release was close to unity, so that low-Ca rigor tension dropped to the zero tension level, and this tension level was maintained until the application of restretch. The complete disappearance of tension recovery was observed over several release-restretch cycles. Similar results were obtained in 10 fibers examined. In some fibers, rigor tension dropped below zero tension baseline for the first and the subsequent releases without exhibiting tension recovery. Some experiments were also made, in which the amplitude release was reduced from 0.5 to 0.3% of Lo, so that T_1_ was well above zero tension baseline. Tension recovery was also absent in this experimental condition (Fig 7C).

## Discussion

### Definite Differences in the Tension Recovery between High-Ca and Low-Ca Rigor Muscle Fibers

In the present experiments, we have shown that, in response to applied ramp-shaped release, the tension in rigor fibers showed not only initial elastic drop, but also subsequent tension recovery (Figs 4, 5, 6 and 7). In high-Ca rigor fibers, the tension recovery was observed over many minutes (Fig 6), and was not appreciably affected by EDTA, while in low-Ca rigor fibers the tension recovery was completely eliminated by EDTA (Fig 7B). These results indicate that, contrary to general view that rigor tension is passively maintained, the AM linkages in rigor fibers exhibit dynamic response to applied releases. As already mentioned in the Results, the time course of tension recovery following ramp-shaped releases in rigor muscle fibers (Figs 4, 6 and 7) resembles that of quick tension recovery following quick release in contracting muscle fibers [15], though the time scale is ~ three orders of magnitude slower in the former than in the latter. In both contracting and rigor fibers, the applied release first produces elastic drop in tension, which is followed by distinct tension recovery to a steady level. The analogy in the time course of tension recovery between contracting and rigor fibers may be taken to indicate that both phenomena may originate from changes in configuration of myosin heads attached to actin during and after applied release. On application of restretch, on the other hand, the tension in rigor fibers rose to a peak and then decayed exponentially, like a viscoelastic system consisting of elastic and viscous elements connected in series. This seems to indicate that, the applied restretch is mainly taken up by viscoelastic sarcomere structures other than attached myosin heads, probably due to nonlinear elasticity of myosin heads with respect to externally applied length changes. The viscoelastic sarcomere structures may include actin and myosin filaments, Z-band and M-line structures. At present we reserve further discussions on this complicated issue.

As expected at the start of the present study, definite differences were found between high-Ca and low-Ca rigor fibers with respect to the tension recovery. In high-Ca rigor fibers, the tension recovery was observed over many minutes (Fig 6), and was not appreciably affected by EDTA. The tension recovery in high-Ca rigor fibers was not affected appreciably by EDTA, while in low-Ca rigor fibers the tension recovery decreased rapidly with time (Fig 7A), and was completely eliminated in the presence of EDTA (Fig 7B). These differences can be accounted in the following way. EDTA chelates free Mg^2+^ions in solution, but can not chelate Mg bound to nucleotide-AM complex.

When muscle fibers, contracting in contracting solution, are transferred into high-Ca rigor solution, a large proportion of myosin heads may be in the state of AMADP, and EDTA can not chelate Mg^2+^ bound to AMADP. Consequently, EDTA has no appreciable effect in reducing the large proportion of AMADP myosin heads, which are responsible for the tension recovery in high-Ca rigor fibers. The long-lasting tension recovery in high-Ca rigor fibers (Fig 6) indicates long lifetimes of AMADP myosin heads. If, on the other hand, muscle fibers in relaxing solution are put into low-Ca rigor solution, EDTA effectively chelates free Mg^2+^ to result in marked reduction in the proportion of AMADP myosin heads in low-Ca rigor fibers.

It is generally believed that, in rigor muscle fibers, all myosin heads form rigor linkages with actin [8]. On this basis, the result that the value of (To–T_1_)/To, representing apparent muscle fiber stiffness, was appreciably larger in low-Ca rigor fibers (≥1, Fig 7) than in high-Ca rigor fibers (<1, Figs 4 and 6) suggests that the apparent stiffness in individual rigor myosin heads is larger in low-Ca rigor fibers than in high-Ca rigor fibers. It seems possible that the value of apparent muscle fiber stiffness may result from past-history dependence of rigor myosin head structure; in high-Ca rigor fibers, myosin heads are expected to bind with actin after performing their last power stroke, while in low-Ca rigor fibers, myosin heads have to override tropomyosin before binding with actin.

**Fig 8:**
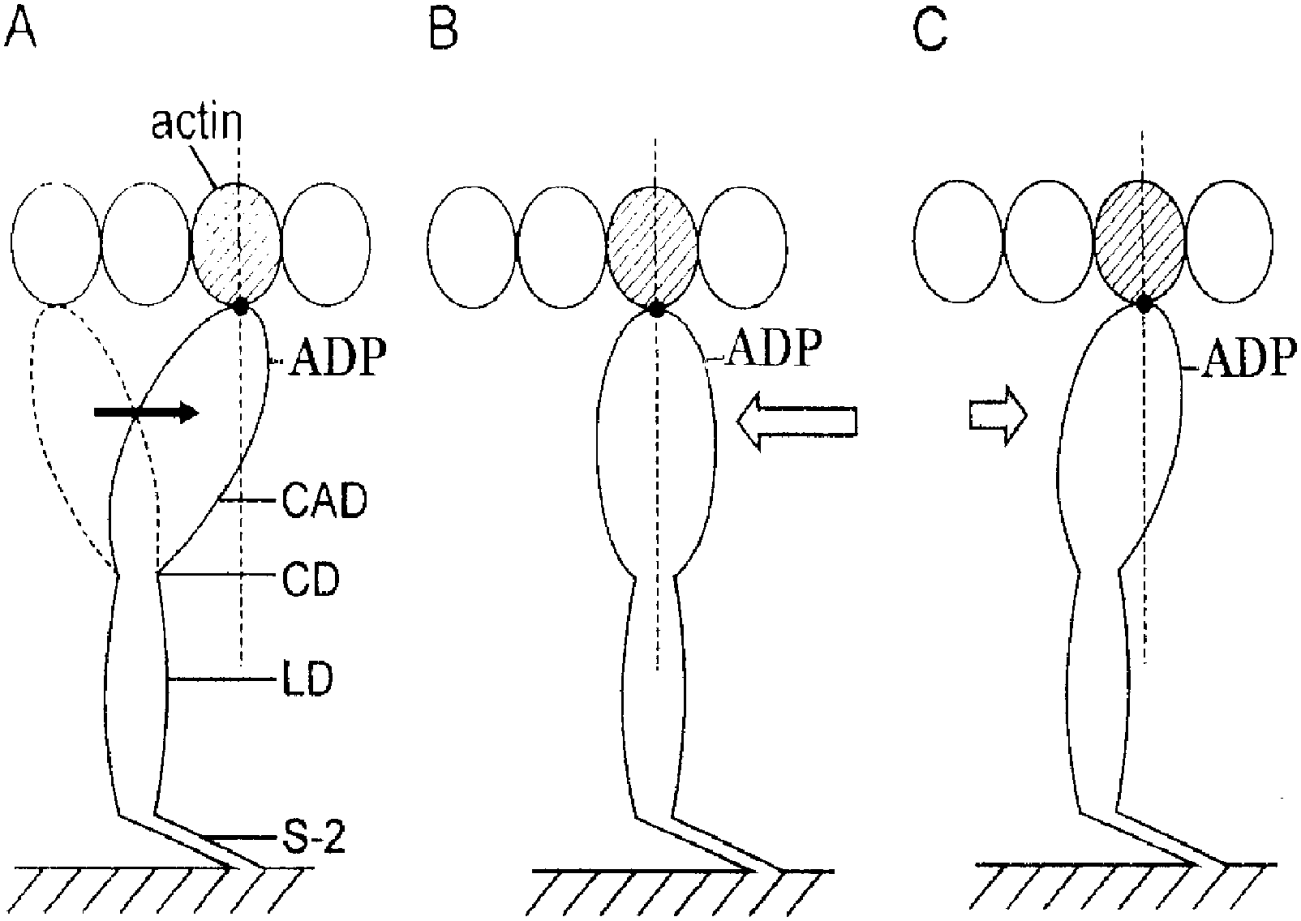
Diagrams showing possible mechanism of tension recovery following release in high-Ca rigor fibers. (A) A myosin head, consisting of catalytic (CAD), converter (CD), and lever arm (LD) domains, extends from myosin filament to attach an actin monomer (shaded) in actin filament, while preserving its configuration at the end of power stroke. Arrow indicated direction of power stroke. (B) On application of release, myosin head is displaced in direction indicated by arrow, producing elastic drop in rigor tension. (C) After completion of release, limited degree of elastic restoration takes place in distorted myosin head, to show up as tension recovery.

Fig 8 shows diagrams illustrating possible mechanism of tension changes in rigor fibers in response to applied release. A myosin head, consisting of catalytic (CAD), converter (CVD) and lever arm (LD) domains, is connected to myosin filament backbone via myosin subfragment-2 (S-2), and, in high-Ca rigor fibers, attaches to actin at the distal region of CAD in the form of AMADP, more or less preserving myosin head configuration at the end of power stroke (Fig 8A). When high-Ca rigor fibers are subjected to ramp-shaped releases, the applied displacement (in the direction of fiber shortening) may mostly be taken up by change in configuration of AMADP myosin heads. To make the matter simple, the myosin head configuration changes are represented as the change in angle of attachment of myosin head to actin, resulting in the elastic tension drop coincident with the applied release (Fig 8B). After completion of release, AMADP myosin heads restore their angle of attachment to actin to a limited extent, resulting in the tension recovery to a steady level (Fig 8C). On this basis, the initial tension drop during applied release and the subsequent tension recovery in high-Ca rigor fibers might correspond to phase 1 and phase 2 in the Huxley-Simmons contraction model [15], respectively. Although it is at present a puzzle why the tension recovery in rigor fibers is ~three orders of magnitude slower than that in contracting fibers, the tension recovery in rigor fibers constitutes evidence for the dynamic nature of AMADP myosin heads, which are present in rigor muscle fibers. Much more experimental work is necessary to explore molecular structure of the AM linkages responsible for muscle contraction. The large variation in half-rise time of tension recovery as well as the variation in the Trec values suggest that the tension recovery may not take place uniformly along the entire length of rigor fibers. For this reason, we reserve further discussions on the origin of tension recovery.

### Implications of the Present Results on the State of Myosin Heads at the End of Power Stroke During Muscle Contraction

As already mentioned, myosin heads in isometrically contracting muscle fibers are supposed to form rigor linkages with actin after performing their last power stroke, when the fibers are transferred to high-Ca rigor solution.

It seems therefore natural to consider that the proportion of AMADP myosin heads, which may correspond to the state of myosin heads at the end of power stroke, is fairly large in high-Ca rigor fibers. On the other hand, the proportion of AM myosin heads is generally believed to be very small in the presence of a high concentration of ATP (~4mM) in the interior of the fiber [7]. If the long life times of AMADP myosin heads, as suggested in the present study (Fig 6), is taken into consideration, the proportion of AM • ADP myosin heads may also be large in contracting muscle. This idea may give answer to the long lasting question why contracting muscles exhibit intermediate X-ray diffraction pattern between those from relaxed and rigor muscles despite a large proportion of myosin heads attached to actin in the form of AMADP and AM [8]; it may be that the AM linkages in contracting muscle are formed with much less transfer of myosin heads onto actin, compared to the AM linkage, determined on extracted protein samples [4,5]. In this connection, it is of interest that Radocaj et al. [16] also present evidence for a new type of AM linkage in contracting muscle fibers based on their X-ray diffraction studies on contracting muscle fibers. Although the structure of the AM linkages, present in contracting muscle, are at present unknown, it seems possible that myosin heads form linkages with actin by some electrostatic mechanism, as suggested by Elliott and his coworkers [17–19]. The idea of electrostatic mechanism is supported by our finding that the force generated by individual myosin heads in Ca^2+^-activated muscle fibers increases ~twofold at low ionic strength, which is expected to strengthen electrostatic myosin head-actin binding [11].

In low-Ca rigor fibers, on the other hand, the proportion of AMADP myosin heads would be small, since myosin heads have to override tropomyosin around actin filaments to bind with actin in various ways. When EDTA is present in relaxing solution, in which formation of AM linkages is in progress, free Mg^2+^ions, required for formation of AMADP myosin heads are quickly removed from low-Ca rigor solution. As the result, low-Ca rigor fibers contain little or no AMADP myosin heads to result in complete elimination of the tension recovery to applied release (Fig 7B). If this explanation is correct, the higher apparent stiffness of low-Ca rigor fibers in the presence of EDTA (Fig 7B) may result from high stiffness of static AM myosin heads, which do not exhibit the tension recovery in response to applied release. The non-dynamic AM myosin heads in low-Ca rigor fibers would gradually change to the AM linkages, determined using extracted protein samples [5]. This view is consistent with the X-ray diffraction pattern from rigor muscle prepared in low-Ca conditions, which shows marked mass transfer of myosin heads to actin [8].
